# A positive psychology group intervention in Greek university students by the counseling center: Effectiveness of implementation

**DOI:** 10.3389/fpsyg.2022.965945

**Published:** 2022-08-24

**Authors:** Kalliope Kounenou, Antonios Kalamatianos, Aikaterini Garipi, Ntina Kourmousi

**Affiliations:** ^1^Department of Education, School of Pedagogical and Technological Education, Athens, Greece; ^2^Student Counselling Centre, School of Pedagogical and Technological Education, Athens, Greece

**Keywords:** positive psychological intervention, positive emotions, resilience, subjective happiness, optimism, self-esteem

## Abstract

Most institutions in higher education have emphasized success in knowledge while overlooking Students’ wellbeing. The present study aimed to examine the effectiveness of the implementation of a 5-week positive psychology group intervention to a sample of 69 students that were assigned to the intervention (*N* = 34) and the control group (*N* = 35). Pre and post measures were taken assessing positive and negative emotions, resilience, happiness, optimism, and self-esteem. In particular, Modified Differential Emotions Scale (mDES), Connor-Davidson Resilience Scale (CD-RISC), Subjective Happiness Scale (SHS), Life Orientation Test-Revised (LOT-R), and Rosenberg Self-Esteem Scale (RSES) were administered to the participants. A mixed measures design was employed with the condition experimental vs. control group as the between-participants factor and time, namely, baseline vs. post-intervention as the within-participants factor. Except for optimism, compared with students in the control group, students in the experimental group showed no significant differences at baseline and experienced a significant increase in positive emotions and resilience in the post-test. On the contrary, the control group demonstrated no significant difference at post-test. Additionally, the students of the intervention group reported significantly higher levels in all measures in comparison with the students of the control group, except resilience, at post-test. However, when the interaction of design and time was considered, the increase in positive emotions solely emerged as a significant result of the intervention. The implementation of a positive psychology group intervention program can be effective in helping students experiencing positive emotions. More research is needed in order to refine and improve the application of such a program in a university setting, in regard to long term intervention.

## Introduction

Over the last two decades, positive psychology interventions have been developed and employed in various settings in order to increase people’s sense of wellbeing. According to the Broaden-and-Build Theory of Positive Emotions (Fredrickson, 1998), experiencing positive emotions broadens and enriches one’s possible alternative ways of thinking and acting. This enlargement results in the acquisition of enduring mental, physical, and social resources, which the individual can use in various situations. In that way, personal growth advances and other positive emotions are fueled, hence leading to a new expansion, thus forming a constantly evolving kind of upward spiral, that fortifies one’s mental health against any disorder, making people more psychologically resilient (Fredrickson et al., 2003), resulting in the experience of new positive emotions, and promoting their wellbeing.

A result of the aforementioned process is the gradual increase in the psychological resilience and emotional wellbeing of individuals (Fredrickson and Joiner, 2002). Resilience refers to the positive adjustment of the individuals despite the difficulties they encounter in being effective and in recovering from stressful experiences (Luthar et al., 2000). People high in psychological resilience have been found to endorse an optimistic view of life and to be open to new experiences and characterized by high positive emotional expression (Grant and Kinman, 2014; Bolton et al., 2017). The relation between positive emotions and psychological resilience has been documented by several researchers (Tugade and Fredrickson, 2002, 2007; Fredrickson et al., 2003).

Moreover, positive emotions have been considered as an ingredient of happiness (Cohn et al., 2009) that is viewed as a state. Experiencing positive emotions, people can gradually build resources and use them in order to meet new challenges Developing this build effect is a key factor that enhances happiness (Cohn et al., 2009).

Furthermore, self-esteem has been defined as “the disposition to experience oneself as competent to cope with the basic challenges of life and as worthy of happiness” by Branden (1994, p. 44). According to Baumeister et al. (2003), high self-esteem leads to greater happiness and is associated with pleasant feelings. Balgiu (2017) and Wang and Kong (2020) have also indicated the relationship between resilience and self-esteem.

Optimism has also been linked with positive affectivity (Scheier and Carver, 1992). Optimism is a trait that has been defined as “…the generalized expectations of the occurrence of good outcomes in one’s life.” (Scheier and Carver, 1985, p. 239) and as “…an individual difference variable that reflects the extent to which people hold generalized favorable expectancies for their future” (Carver et al., 2010, p. 879). Brissette et al. (2002) have noticed relations between optimism and distress in students starting college.

Positive Psychological Interventions (PPIs) are theoretically grounded and empirically validated instructions, activities, and recommendations designed to enhance wellbeing (Lomas et al., 2014). PPIs are low cost interventions, easy to deliver, while being non-stigmatizing and lacking side-effects (Layous et al., 2011). PPI strategies that have manifested an improvement in wellbeing contain writing gratitude letters, practicing optimistic thinking, replaying positive experiences, and socializing (Bolier et al., 2013). Pioneers in this field (Fordyce, 1977, 1988) found that happiness levels could be increased by “shotgun” interventions involving multiple exercises. As reported by Lyubomirsky (2007), PPIs, that include consistent gratitude practice, have a positive correlation with greater happiness, more energy, more hope toward the future, and more positive emotions. It has also been demonstrated that people who tend to perform acts of kindness are more likely to describe themselves as happy (Lyubomirsky et al., 2004).

Undergraduate students often experience challenges, as the University life is a period of transition from being a teenager to young adulthood, in which they need to learn to adjust and cope with changes in their everyday life (Arnett, 2000; Trigueros et al., 2020). Hence, positive emotions, e.g., feelings of happiness, could help improve a Student’s resilience (Tugade and Fredrickson, 2007) and serve as a determinant of his/her psychological need satisfaction (Howell et al., 2011) and psychological wellbeing (Hasnain et al., 2014).

Despite relevant evidence about the benefits of positive education, higher education is frequently left out of discussions involving the implementation of wellbeing skills (Oades et al., 2011; Norrish et al., 2013). Academic institutions have always put emphasis on acquisition and excellence regarding knowledge and exam performance. Nevertheless, there is an elevated interest in people’s wellness within the academic environment (Lambert et al., 2019), especially after the covid-19 quarantine implementation and during the post-covid-19 era. In Greece, PPIs that focus on a single component or a combination of them have proven to be effective (Pezirkianidis and Stalikas, 2020). However, there are only a few studies about PPIs that have been applied in educational settings, such as a 4-week study aiming at promoting wellbeing in a multicultural school setting by Dimitropoulou and Leontopoulou (2017) and a 4-week on-line program enhancing self-compassion, positive emotion, and mental resilience, while reducing isolation, over-identification, self-judgment, trait anxiety, and state anxiety (Karakasidou et al., 2021).

In the current study, the Counseling Centre of Pedagogical and Technological Education (ASPETE) tried to address this gap by offering evidence of a PPI program delivered to a group of university students in Greece. Overall, in keeping with the benefits of this approach to higher education and with the shift of the perspective away from student weaknesses and toward the recognition of their strengths (Williams et al., 2018), the Counseling Centre investigated core concepts of positive psychology among the students, such as experiencing positive emotions and psychological resilience, together with subjective happiness, optimism, and self-esteem. Implementing a widely used group PPI, rather than viewing students as flawed, targeted these major positive psychology concepts and attempted to help students recognize their existing resources and build their strengths (Hefferon and Boniwell, 2011).

## Aim and hypotheses

The aim of this study was to investigate whether a group PPI program among university students can increase positive emotions, resilience, subjective happiness, optimism, and self-esteem, and decrease negative emotions. To our knowledge the relationship of the aforementioned factors in terms of a positive psychology, psycho-educational group program has not been examined in Greek university students. In particular, we expected the following:

Students in the experimental group will report significantly increased levels of positive emotions, resilience, optimism, subjective happiness, and self-esteem, and significantly decreased levels of negative emotions between baseline and post-test.

Students in the control group will report no significantly increased levels of positive emotions, resilience, optimism, subjective happiness, and self-esteem, and no significantly decreased levels of negative emotions between baseline and post-test.

Students in the experimental group will report significantly increased levels of positive emotions, resilience, optimism, subjective happiness, and self-esteem, and significantly decreased levels of negative emotions compared to students in the control group at post-test.

## Materials and methods

### Participants

Sixty-nine individuals, 52 men and 17 women were recruited from the three Engineering departments of ASPETE. Participants were informed about the purpose of the study and gave their informed consent. The individuals received no reward for their participation. The average age was 22.09 years (*SD* = 6.52) for the experimental group and 20.97 years (*SD* = 0.99) for the control group. With regard to sex, the experimental group included 22 males (64.7%) and 12 females (35.3%) and the control group consisted of 30 males (85.7%) and 5 females (14.3%). The Chi-square test revealed significant difference in the two groups (χ^2^ = 4.10, *p* < 0.05), though of weak effect size (Cramer’s *V* = 0.24).

### Design and procedure

We employed a mixed measures design. The between-participants factor was the condition experimental vs. control group and the within-participants factor was time, namely, baseline vs. post-intervention/follow up. Hence, we recruited a convenience sample of 75 students from civil and mechanical engineering educators’ classes of ASPETE, for the experimental group and 89 students from electrical engineering educators’ courses for the control group. The study was conducted in accordance with the Declaration of Helsinki ethical principles. In order to assure confidentiality and anonymity we used personal codes for every participant.

Initially, both groups completed *in vivo* the following questionnaires: The Differential Emotions Scale-modified (mDES), the Connor-Davidson Resilience Scale (CD-RISC), the Subjective Happiness Scale (SHS), the Revised Life Orientation Test (LOT-R), and the Rosenberg Self-Esteem Scale (RSES).

The positive psychology group-counseling intervention consisted of five 1.5–2 h psychoeducational seminars offered across 5 weeks to the students. The techniques used were derived from different positive psychology intervention programs offered to young adults, like, mostly, the 8-week positive psychotherapy group by Parks and Seligman (2007), the how of happiness (Lyubomirsky, 2007), and the Acts of Kindness interventions (Aok) (Lyubomirsky et al., 2005).

More specifically, in the experimental group, in each session two psychologists provided a brief lecture to introduce new material and, later on, the students completed forms related to their everyday experience in each session. In the end of each session they were handed worksheets with the week’s exercise that concerned the recording of various aspects of their experience. The participants were urged to discuss their answers. The group program entailed various exercises, such as the three good things, the letter of gratitude, using one’s strengths, savoring, kindness, and life summary, tailored to the Students’ needs (Table 1). No intervention was provided to the participants of the control group. At the end of the program students of both groups were re-administered *in vivo* the measures. In the experimental group, 41 individuals were excluded because they did not fully attend the program, whereas in the control team 54 individuals were lost to post-test.

**TABLE 1:** Description of the PPI program in every session.

Session	Topic	Content	Tasks/exercises	Homework
1	Introduction and initial measurement	Information about the aim of the intervention, administration of questionnaires	Savoring and mindfulness Exercises regarding automatism	No
2	Positive psychology and positive feelings	Information about positive psychology and main topics, how it can help students, aim of the Three Good Things exercise	Three good things	Three good things every day in a weekly span
3	Gratitude	Theory about gratitude and its benefits (Emmons and McCullough, 2003)	Questionnaire about gratitude, three things I am grateful for, letter of gratitude	Journal of gratitude for a week
4	Character strengths	Presentation of virtues and character strengths (Peterson and Seligman, 2004)	Questionnaire about gratitude	List of accomplishments and achievements
	Act of kindness	Act of kindness, meaning and aim (Lyubomirsky et al., 2004)	Life summary	5 Act of kindness in 1 day Act of kindness toward self
5	Ending and final measurement	Summary of the program, reflection on students’ experience, re-administration of questionnaires	Personal motto	No

### Measures

Basic demographic information included gender, age, and faculty.

Emotions were measured by the Modified Differential Emotions Scale-mDES constructed by Izard (1977) and modified by Fredrickson (2009). It is used to assess specific distinct, positive and negative emotions the respondents experience within the last 2 weeks. The test includes 21 questions and evaluates 11 positive and 9 negative emotions. The last question refers to the emotion experienced most intensely by each participant. The answers are given on a five-point Likert scale (1 “*Not at all*,” 5 “*Extremely*”). Its Greek standardization revealed satisfactory reliability (Galanakis et al., 2016). Cronbach’s alphas of the Positive Emotions subscale and the Negative Emotions subscales in the present study were 0.817 and 0.840, respectively.

Resilience was measured by the Connor-Davidson Resilience Scale (CD-RISC; Connor and Davidson, 2003). It assesses one’s ability to surpass the adversities and specifically, tenacity and competence, trusting in one’s instincts and tolerating of negative affect, accepting of change and security within relationships, control, and spirituality. It consists of five factors, namely, personal competence, tolerance, acceptance of change, control, and spiritual influences, and contains 25 items (“I am able to adapt when changes occur”). Responses range from: 0—*Not true at all* to 4—*True nearly all the time* and higher scores is an indicator of high resilience. It exhibits good psychometric properties. Cronbach’s alpha of CD-RSIC in this study was 0.863.

Happiness was assessed by the Subjective Happiness Scale-SHS (SHS; Lyubomirsky and Lepper, 1999). Ratings are made on a 7-point Likert scale (1 “*Not a very happy person*” to 7 “*A very happy person*”) and higher scores reflect greater happiness (e.g., “Some people are generally very happy. They enjoy life regardless of what is going on, getting the most out of everything. To what extent does this characterization describe you?”). Its Greek standardization (Karakasidou et al., 2016) has shown that Cronbach’s alpha index was 0.76 and the split half reliability index was 0.72. As for criterion validity, the SHS was significantly negatively correlated to negative emotions, stress, and depression and positively correlated to life satisfaction, resilience, and positive emotions. Cronbach’s alpha of 0.842 was obtained in this study.

Optimism was assessed by the Life Orientation Test-LOT-R that identifies one’s dispositional level of optimism. It was created by Scheier and Carver (1985) and modified by Scheier et al. (1994). It consists of ten statements (“Overall, I expect more good things to happen to me than bad”) rated on a 5-grade Likert scale ranging from 0 “*Strongly disagree*” to 4 “*Strongly agree.*” The respondents express their general expectations regarding future outcomes. LOT-R has shown good psychometric properties. Coefficient alpha reliability for the LOT-R was 0.78 and convergent and discriminant validity were demonstrated when the LOT-R correlated positively with measures of self-mastery and the self-esteem and negatively with a measure of anxiety. It has been adapted to Greek by Lyrakos et al. (2010). Cronbach’s alpha of 0.717 was obtained in this study.

Self-esteem was measured by the RSES (Rosenberg et al., 1995). It contains 10 items (“On the whole, I am satisfied with myself”) and is used to assess global self-esteem. Respondents are asked to rate on a 4-point Likert scale ranging from 1 “*Strongly agree*” to 4 “*Strongly disagree*” and higher scores indicate more positive self-regard. As for reliability, test-retest correlations values are 0.82 and 0.88. In its Greek validation the internal consistency reliability index was found 0.80 (Galanou et al., 2014). RSES has also exhibited good concurrent, predictive, and construct validity (Rosenberg, 1979). Cronbach’s alpha of RSES in the present study was 0.823.

### Statistical analysis

Initially we tested for linearity with the scatter plots and found no deviation. We also checked for normality with histograms, Q-Q plots, goodness of fit tests, e.g., the Kolmogorov-Smirnov and the Shapiro-Wilk test, and skewness and kurtosis values and no statistically significant violations were found (Sposito et al., 1983; Orcan, 2020). Correlations between the measured variables and means, standard deviations, and *t*-tests at baseline and post-intervention were calculated. A MANOVA was conducted to compare positive and negative emotions, resilience, subjective happiness, optimism, and self-esteem in positive intervention and no positive intervention condition prior to the program. Moreover, a paired-samples *t*-test was conducted to compare positive and negative emotions, resilience, subjective happiness, optimism, and self-esteem in each group, at two time points, baseline and post-test. Finally, mixed ANOVAs were performed with the experimental condition as the between-subjects variable and time (baseline-post-test) as the within-participants factor so as to examine the effect of the intervention. SPSS, version 21, was used.

## Results

The majority of the correlations were positive and statistically significant except the non-significant relations of post-test negative emotions with pre-test positive emotions and self-esteem. Pre and post-test negative emotions demonstrated negative correlations with all the measures. The strongest correlation was found between post-test positive emotions and resilience (Table 2).

**TABLE 2:** Correlations between measured variables.

	PosEmP	NegEmP	PosEmPo	NegEmPo	CD-RISCP	CD-RISCPo	SHSP	SHSPo	LOT-RP	LOT-RPo	RSESP	RSESPo
PosEmotionP	1											
NegEmP	−0.422**	1										
PosEmotionPo	0.570**	−0.347**	1									
NegEmPo	−0.178*ns*	0.599**	−0.520**	1								
CD-RISCP	0.663**	−0.501**	0.529**	−0.461**	1							
CD-RISCPo	0.485**	−0.368**	0.735**	−0.556**	0.712**	1						
SHSP	0.613**	−0.590**	0.521**	−0.444**	0.627**	0.492**	1					
SHSPo	0.417**	−0.390**	0.697**	−0.526**	0.422**	0.674**	0.546**	1				
LOT-RP	0.399**	−0.440**	0.444**	−0.416**	0.517**	0.492**	0.655**	0.524**	1			
LOT-RPo	0.386**	−0.530**	0.484**	−0.536**	0.498**	0.624**	0.581**	0.592**	0.678**	1		
RSESP	0.504**	−0.287*	0.279*	−0.154*ns*	0.424**	0.379**	0.330**	0.505**	0.502**	0.436**	1	
RSESPo	0.292*	−0.468**	0.574**	−0.669**	0.479**	0.576**	0.418**	0.536**	0.398**	0.611**	0.328**	1

P, Pre-intervention; Po, Post-intervention; PosEm, Modified Differential Emotions Scale Positive Emotions; NegEm, Modified Differential Emotions Scale Negative Emotions; CD-RISC, Connor-Davidson Resilience Scale; SHS, Subjective Happiness Scale; LOT-R, Life Orientation Test-Revised; RSES, Rosenberg Self-Esteem Scale. *p < 0.05, **p < 0.01, ns, non-significant.

Equivalence at baseline was checked with MANOVA. The independent variable was the two level (experiment vs. control) condition and the dependent variables were all the measures. The participants who received the positive psychology intervention (*M* = 3.55, *SD* = 0.671, *M* = 1.79, *SD* = 0.561, *M* = 2.77, *SD* = 0.509, *M* = 5.07, *SD* = 0.930, *M* = 3.01, *SD* = 0.620) compared to the participants in the control group (*M* = 3.41, *SD* = 0.650, *M* = 2.04, *SD* = 0.809, *M* = 2.70, *SD* = 0.427, *M* = 4.57, *SD* = 1.232, *M* = 2.96, *SD* = 0.487) did not demonstrate significantly better positive emotions scores, *F*(1, 67) = 0.770, *p* = 0.383, $\eta_{p}^{2}$ = 0.011, negative emotions scores, *F*(1, 67) = 2.256, *p* = 0.138, $\eta_{p}^{2}$ = 0.033, resilience, *F*(1, 67) = 0.450, *p* = 0.505, $\eta_{p}^{2}$ = 0.007, subjective happiness, *F*(1, 67) = 3.531, *p* = 0.065, $\eta_{p}^{2}$ = 0.050, and self-esteem, *F*(1, 67) = 0.149, *p* = 0.700, *$\eta_{p}^{2}$* = 0.002, correspondingly. A statistically significant difference was revealed only for optimism, *F*(1, 67) = 6.555, *p* < 0.05, $\eta_{p}^{2}$ = 0.089, where individuals who attended the positive psychology program (*M* = 2.33, *SD* = 0.699) scored higher than the students in the control group (*M* = 1.91, *SD* = 0.660).

With regard to within-subjects differences, we used dependent *t*-test. In the experimental group, the results from the pre-design and post-design measures indicate that the PPI resulted in a statistically significant improvement in positive emotions, *t*(33) = −3.479, *p* < 0.01, resilience, *t*(33) = −2.752, *p* < 0.05, and marginally in self-esteem, *t*(33) = −1.962, *p* = 0.058, and not significant differences in negative emotions, *t*(33) = 1.479, *p* = 0.149, subjective happiness, *t*(33) = −0.850, *p* = 0.401, and optimism, *t*(33) = −1.489, *p* = 0.146 (Table 3).

**TABLE 3:** Means, standard deviations, and pre and post-intervention between-groups and within-groups differences.

Pre-test	Experimental group				Control group				Independent *t*-test	
			
	*M*	*SD*			*M*	*SD*				Sig
Positive emotions	3.55	0.671			3.41	0.650			0.877	0.383 ns
Negative emotions	1.79	0.561			2.04	0.809			−1.502	0.138 ns
CD-RISC	2.77	0.509			2.70	0.427			0.671	0.505 ns
SHS	5.07	0.930			4.57	1.232			1.879	0.065 ns
LOT-R	2.33	0.699			1.91	0.660			2.560	<0.05
RSES	3.01	0.620			2.96	0.487			0.386	0.700 ns

**Post-test**	**Experimental group**				**Control group**					
				
			**Paired *t*-test**				**Paired *t*-test**		**Independent *t*-test**	
							
	***M***	***SD***		**sig**	***M***	***SD***		**sig**		**sig**

Positive emotions	3.88	0.631	−3.479	<0.01	3.37	0.655	0.376	0.709 ns	3.276	<0.01
Negative emotions	1.67	0.480	1.479	0.149 ns	2.05	0.609	−0.125	0.901 ns	−2.934	<0.01
CD-RISC	2.96	0.509	−2.752	<0.05	2.73	0.496	−0.560	0.579 ns	1.859	0.067 ns
SHS	5.23	1.089	−0.850	0.401 ns	4.54	1.026	0.167	0.868 ns	2.689	<0.01
LOT-R	2.48	0.685	−1.489	0.146 ns	2.08	0.663	1.823	0.077 ns	2.490	<0.05
RSES	3.23	0.482	−1.962	0.058 ns	2.92	0.482	0.453	0.653	2.691	<0.01

Sig, significance; ns, non-significant; Pre-test, Pre-intervention; Post-test, Post-intervention; Positive Emotions, Modified Differential Emotions Scale Positive Emotions; Negative Emotions, Modified Differential Emotions Scale Negative Emotions; CD-RISC, Connor-Davidson Resilience Scale; SHS, Subjective Happiness Scale; LOT-R, Life Orientation Test-Revised; RSES, Rosenberg Self-Esteem Scale.

As for the dependent *t*-test in the control group, the results from the pre-design and post-design measures resulted in no statistically significant difference in positive emotions, *t*(34) = 0.376, *p* = 0.709, negative emotions, *t*(34) = −0.125, *p* = 0.901, resilience, *t*(34) = −0.560, *p* = 0.579, subjective happiness, *t*(34) = 0.167, *p* = 0.868, optimism, *t*(34) = 1.823, *p* = 0.077, and self-esteem, *t*(34) = 0.453, *p* = 0.653 (Table 3).

Finally, a series of mixed ANOVAs revealed several statistically significant differences. In particular, we found significant interaction between condition and time for positive emotions, *F*(1, 67) = 6.463, *p* < 0.05, $\eta_{p}^{2}$ = 0.088. However, negative emotions, *F*(1, 67) = 0.924, *p* = 0.340, $\eta_{p}^{2}$ = 0.014, resilience, *F*(1, 67) = 2.827, *p* = 0.097, $\eta_{p}^{2}$ = 0.040, subjective happiness, *F*(1, 67) = 0.556, *p* = 0.459, $\eta_{p}^{2}$ = 0.008, optimism, *F*(1, 67) = 0.012, *p* = 0.915, $\eta_{p}^{2}$ = 0.000, and self-esteem, *F*(1, 67) = 3.207, *p* = 0.078, $\eta_{p}^{2}$ = 0.046 were not significantly affected by the intervention. Thus, students in the experimental group reported less significant negative emotions (*M* = 1.67, *SD* = 0.631), not significantly higher resilience (*M* = 2.96, *SD* = 0.509), significantly higher subjective happiness (*M* = 5.23, *SD* = 1.089), optimism (*M* = 2.48, *SD* = 0.685), and self-esteem (*M* = 3.23, *SD* = 0.482) as compared with participants in the control group (*M* = 2.05, *SD* = 0.609, *M* = 2.73, *SD* = 0.496, *M* = 4.54, *SD* = 1.026, *M* = 2.08, *SD* = 0.663, *M* = 2.92, *SD* = 0.482), respectively (Table 3), but not due to the intervention. On the contrary, the significant increase of positive emotions in the experimental group (*M* = 3.88, *SD* = 0.631) with comparison to the control group (*M* = 3.37, *SD* = 0.655) seems to be due to the intervention.

## Discussion

The aim of this study was to investigate whether a PPI program among university students can be effective as well as to provide evidence concerning the dynamics of positive psychology in higher education and evaluate the interplay between positive and negative emotions, resilience, subjective happiness, optimism, and self-esteem.

Initially, it was confirmed that students in both groups showed no significant difference in their scores regarding all the measures before the intervention. This is in line with previous studies (Luthans et al., 2008). However, participants in the experimental group demonstrated higher scores in optimism at pre-test.

Results also indicated that the intervention led the students of the PPI program to a significant improvement in positive emotions, resilience and slightly in self-esteem. This result is in agreement with previous studies that have suggested that being emotionally positive could increase the level of resilience (Kay, 2016). Positive emotions are one of the key mechanisms which can influence psychological and physical help (Moskowitz et al., 2014). Moskowitz et al. (2021), in their review on the effects of PPIs, have found that PPIs have significant influence on positive emotion, especially when we examine the change within the intervention group or when we evaluate the intervention vs. a minimum control condition such as a waitlist. In another study by Lambert et al. (2021) college students in the United Arab Emirates, who participated in a PPI program, reported more positive emotions and an overall balance of feelings that favored positivity over time in comparison to the control group. Nevertheless, after the intervention, the experimental group showed no statistically significant difference in negative emotions, subjective happiness, and optimism, contrary to other studies, such as the research by Huang et al. (2016), that demonstrated improvements in participants’ optimism, compared to a control group. However, similar results were found by Joutsenniemi et al. (2014) with a large community sample of Finnish adults, who discovered that an intervention constituting of gratitude, optimism, and rumination reduction showed no significant improvement in levels of happiness.

A growing body of evidence has noted that happiness-increasing strategies do stimulate elevation in wellbeing and happiness. A meta-analysis of 51 positive interventions overwhelmingly revealed that positive interventions significantly increase wellbeing and alleviate depressive symptoms (Sin and Lyubomirsky, 2009). This was not confirmed in our study and it can be attributed to different factors. In particular, when it comes to psychological research, there is a big number of examples in the psychological, educational, and health literature of interventions that have been successful in one place or at one time, but failed to produce similar effects in another place or another time, despite having implemented the original successful protocol (Bohrnstedt and Stecher, 2002; Cartwright and Hardie, 2012). We should bear in mind that the results of an intervention may occur in the beginning of the study, but they may also take more time to become obvious, as individuals may assign new meaning to the intervention, due to changes in their lives (Peuker et al., 2009.) Finally, people, who participate in a PPI program and seek ways to improve their wellness, do not form a single, homogeneous group and, thus, individuals may differ in their responses to PPIs depending on their symptoms (Parks et al., 2012). As PPIs have shown small to moderate effect sizes in a methodologically sound meta-analysis (Bolier et al., 2013), more effort should be put into the study of theoretically based moderating variables. However, other researchers have shown that psychological interventions are more efficient for individuals with higher levels of positive affect or more effective emotion regulation strategies, as positive emotions facilitate openness to experience and engage toward meaningful goals (Taylor et al., 2017). Besides, according to a meta-analysis by Carr et al. (2020), PPIs were more effective when they included multiple components, more sessions, and lasted longer.

While our results may be interesting, there are some limitations that should be mentioned. Our sample size was rather small. Moreover, a non-randomized quasi experimental design was used and we did not match the two groups. Our results might not be generalizable to other groups with different ethnic characteristics or academic backgrounds. However, our study has several advantages and implications as well. The existence of an experimental and a control group is a notable strength in the internal validity of the study. We statistically controlled for and assured equivalence at baseline for all variables in the study, except optimism. Further, since one intervention may not fit for all, the current study embedded different positive psychology components in order to increase the chance of a productive outcome (Salois, 2021). What’s more, the counseling centre’s study adds to the growing literature pertaining to PPIs in academic environment. Even so, future research could use randomized trials and a greater sample size, more representative of the Greek university population, from various faculties, e.g., social sciences. We also encourage future studies to use longer time frame with multiple follow-up sessions, since this could help in discovering if the positive outcomes are long lasting, and to associate the positive psychology components with Students’ mental health improvement, achieved grade, and academic self-efficacy.

On the whole, the counseling centre’s research realized for the PPI group important amelioration in optimism, subjective happiness, and self-esteem, reduction in negative emotions and slight increase in resilience. These changes were not observed in the control group and can, thus, be attributed to the intervention, with caution, since, the results suggest that university students significantly increased only positive emotions due to the PPI program they attended. The use of PPI programs in higher education is a relatively new development that could help the field of education. Consequently, higher educational institutions, not only in Greece but globally, could benefit from applying interventions as the one described in our study and see positive changes in students.

## Data availability statement

The raw data supporting the conclusions of this article will be made available by the authors, without undue reservation.

## Ethics statement

Ethical review and approval was not required for the study on human participants in accordance with the local legislation and institutional requirements. The patients/participants provided their written informed consent to participate in this study.

## Author contributions

KK, AK, and AG conceptualized the intervention, wrote the manuscript, and reviewed the manuscript. NK provided the final revision. All authors approved the submission of the manuscript.
